# *Anaplasma phagocytophilum* invasin AipA interacts with CD13 to elicit Src kinase signaling that promotes infection

**DOI:** 10.1128/mbio.01561-24

**Published:** 2024-09-26

**Authors:** Mary Clark H. Lind, Waheeda A. Naimi, Travis J. Chiarelli, Tavis Sparrer, Mallika Ghosh, Linda Shapiro, Jason A. Carlyon

**Affiliations:** 1 Department of Microbiology and Immunology, Virginia Commonwealth University Medical Center, School of Medicine, Richmond, Virginia, USA; 2 Center for Vascular Biology, University of Connecticut School of Medicine, Farmington, Connecticut, USA; 3 Department of Cell Biology, University of Connecticut School of Medicine, Farmington, Connecticut, USA; University of Nebraska Medical Center, Omaha, Nebraska, USA

**Keywords:** *Anaplasma*, invasin, CD13, Src, Syk, obligate intracellular bacteria, *Anaplasmataceae*, *Rickettsiales*, host-pathogen interactions, bacterial internalization

## Abstract

Host-microbe interactions that facilitate entry into mammalian cells are essential for obligate intracellular bacterial survival and pathogenesis. *Anaplasma phagocytophilum* is an obligate intracellular bacterium that invades neutrophils to cause granulocytic anaplasmosis. The invasin-receptor pairs and signaling events that induce *Anaplasma* uptake are inadequately defined. *A. phagocytophilum* invasion protein A orchestrates entry via residues 9–21 (AipA_9–21_) engaging an unknown receptor. Yeast two-hybrid screening suggested that AipA binds within C-terminal amino acids 851–967 of CD13 (aminopeptidase N), a multifunctional protein that, when crosslinked, initiates Src kinase and Syk signaling that culminates in endocytosis. Co-immunoprecipitation validated the interaction and confirmed that it requires the AipA N-terminus. CD13 ectopic expression on non-phagocytic cells increased susceptibility to *A. phagocytophilum* infection. Antibody blocking and enzymatic inhibition experiments found that the microbe exploits CD13 but not its ectopeptidase activity to infect myeloid cells. *A. phagocytophilum* induces Src and Syk phosphorylation during invasion. Inhibitor treatment established that Src is key for *A. phagocytophilum* infection, while Syk is dispensable and oriented the pathogen-invoked signaling pathway by showing that Src is activated before Syk. Disrupting the AipA-CD13 interaction with AipA_9–21_ or CD13_781–967_ antibody inhibited Src and Syk phosphorylation and also infection. CD13 crosslinking antibody that induces Src and Syk signaling restored infectivity of anti-AipA_9–21_-treated *A. phagocytophilum*. The bacterium poorly infected CD13 knockout mice, providing the first demonstration that CD13 is important for microbial infection *in vivo*. Overall, *A. phagocytophilum* AipA_9–21_ binds CD13 to induce Src signaling that mediates uptake into host cells, and CD13 is critical for infection *in vivo*.

**IMPORTANCE:**

Diverse microbes engage CD13 to infect host cells. Yet invasin-CD13 interactions, the signaling they invoke for pathogen entry, and the relevance of CD13 to infection *in vivo* are underexplored. Dissecting these concepts would advance fundamental understanding of a convergently evolved infection strategy and could have translational benefits. *Anaplasma phagocytophilum* infects neutrophils to cause granulocytic anaplasmosis, an emerging disease for which there is no vaccine and few therapeutic options. We found that *A. phagocytophilum* uses its surface protein and recently identified protective immunogen, AipA, to bind CD13 to elicit Src kinase signaling, which is critical for infection. We elucidated the AipA CD13 binding domain, which CD13 region AipA engages, and established that CD13 is key for *A. phagocytophilum* infection in vivo. Disrupting the AipA-CD13 interaction could be utilized to prevent granulocytic anaplasmosis and offers a model that could be applied to protect against multiple infectious diseases.

## INTRODUCTION

Obligate intracellular pathogens, which must invade host cells to survive and cause disease, pose significant threats to human and veterinary health worldwide. Deciphering the microbial invasin-host cell receptor interactions that mediate entry is fundamental to understanding these organisms’ pathogenesis and can reveal opportunities for preventing or treating infection. *Anaplasma phagocytophilum* is a tick-transmitted obligate intracellular bacterium that colonizes peripherally circulating neutrophils to cause granulocytic anaplasmosis in humans, horses, and companion animals and tickborne fever in sheep, cattle, and other ruminants. It is endemic or potentially endemic in 42 countries in North America, South America, Europe, Asia, and Africa (1). Human granulocytic anaplasmosis (HGA) can range from mild infection to severe disease requiring intensive care. HGA symptoms and potential complications include fever, leukopenia, thrombocytopenia, rhabdomyolysis, transaminitis, splenomegaly, increased susceptibility to secondary infections, multiorgan failure, sepsis, shock, and death. Outcomes are more severe for the elderly, immunosuppressed, and when antibiotic treatment is delayed (1, 2). In recent years, the emergence rate of HGA in the United States surpassed that of all other tickborne diseases (3).

*A. phagocytophilum* cycles between an infectious dense-cored (DC) form that invades host cells and a noninfectious reticulate cell (RC) form that replicates intracellularly (4). The DC morphotype orchestrates its receptor-mediated endocytic uptake into a multivesicular body (MVB)-like parasitophorous organelle called the ApV (*A. phagocytophilum*-occupied vacuole) via interactions between *Anaplasma* surface proteins and host cell receptors that are incompletely defined (4–11). The invasion process is caveolae-dependent (12), signifying that at least some of the receptors are in lipid rafts. Each entry event forms a single ApV that matures along the MVB-exocytosis pathway allowing for DC-to-RC conversion, RC binary fission, RC-to-DC reconversion, and release of infectious DC progeny (13, 14). The mammalian host cell signaling events that are essential for *A. phagocytophilum* entry and how the pathogen elicits them are poorly understood (15–17).

Three *A. phagocytophilum* surface proteins that mediate infection have been identified: OmpA (outer membrane protein A), Asp14 (14 kDa *A*. *phagocytophilum* surface protein), and AipA (*A. phagocytophilum* invasion protein A). All are upregulated during RC-to-DC conversion and tick transmission feeding (8, 9, 11). Their receptor binding domains have been delimited as linear stretches of 12–15 amino acids (10, 11). Using antibodies to simultaneously target multiple binding domains synergistically blocks *A. phagocytophilum* infection of host cells and severely reduces the bacterial load *in vivo* (6, 10, 18). OmpA binds the sialyl Lewis x (sLe^x^) glycan cap on P-selectin glycoprotein ligand-1 (PSGL-1) together with an unidentified outer membrane protein (OMP) that binds the PSGL-1 N-terminus to dock the bacterium to the cell surface (5, 7, 9, 10). Asp14 interacts with protein disulfide isomerase (PDI) to promote PDI-mediated reduction of *A. phagocytophilum* cell surface disulfide bonds, which is important for bacterial uptake (6, 8, 10). Like Asp14, AipA is critical for invasion. The 36.9 kDa protein is exclusive to *A. phagocytophilum*. AipA residues 1–87 (AipA_1–87_) constitute an extracellular domain that in recombinant form binds to host cells (11). Immunization of mice against AipA_9–21_ elicits protective immune responses, indicating that the invasin is key for *in vivo* infection and that AipA_9–21_ is an important immunogen (18). The AipA receptor and mechanism by which AipA mediates *A. phagocytophilum* entry into host cells are unknown.

CD13 (aminopeptidase N) plays roles in defense against pathogens, inflammation, antigen presentation, angiogenesis, tumor cell invasion, and metastasis (19–21). It is a glycosylated dimeric transmembrane ectopeptidase that predominantly localizes to caveolae and is expressed on several tissues and cell types. Among hematopoietic cells, CD13 is expressed on stem cells and most developmental stages of myeloid cells, including neutrophils (19). The seahorse-shaped molecule has a seven-domain organization consisting of an 8- to 10-amino acid N-terminal cytoplasmic portion (domain I), a transmembrane domain (II), and a large extracellular region (domains III-VII) that contains the active site. CD13 forms a head-to-head homodimer through interactions between each monomer’s domain VII. It cleaves N-terminal neutral amino acids of small peptides and the N-termini of cytokines, hormones, and chemokines (19, 20). When engaged by an antibody or viral ligand, CD13 performs functions independent of its enzymatic activity including signal transduction that leads to caveolae-dependent endocytosis, phagocytosis, adhesion, or cell migration (19, 22–25). CD13 functionally interacts with Fcγ receptors to enhance phagocytosis but can also mediate uptake without a co-receptor. Hence, it can directly activate intracellular signaling (23, 25, 26). CD13 crosslinking activates Src kinase, spleen tyrosine kinase (Syk), and components of the phosphatidylinositol-3-kinase (PI3K)/Akt pathway, among others (19). CD13 is a receptor for entry by human coronaviruses (CoVs) and cytomegalovirus (CMV), porcine deltaCoV, porcine respiratory CoV, porcine epidemic diarrhea virus, porcine transmissible gastroenteritis virus, feline enteric CoV, feline infectious peritonitis virus, canine CoV, and *Mycobacterium tuberculosis* (19, 27–36). It was also recently identified as a receptor for enterotoxigenic *Escherichia coli* F4 fimbriae to adhere to intestinal epithelial cells (37).

In this study, we identify CD13 as the AipA receptor. The interaction mediated between AipA_9–21_ and CD13 C-terminal residues 851–967 stimulates Src and downstream Syk signaling independent of CD13 peptidase activity. *A. phagocytophilum* requires Src but not Syk activity to infect myeloid cells and CD13 to productively infect mice. Overall, we delineate the first intracellular bacterial invasin that targets CD13, confirm its binding domain, and establish the receptor’s relevance for *in vivo* infection.

## RESULTS

### CD13 is an AipA interacting partner

**Fig 1:**
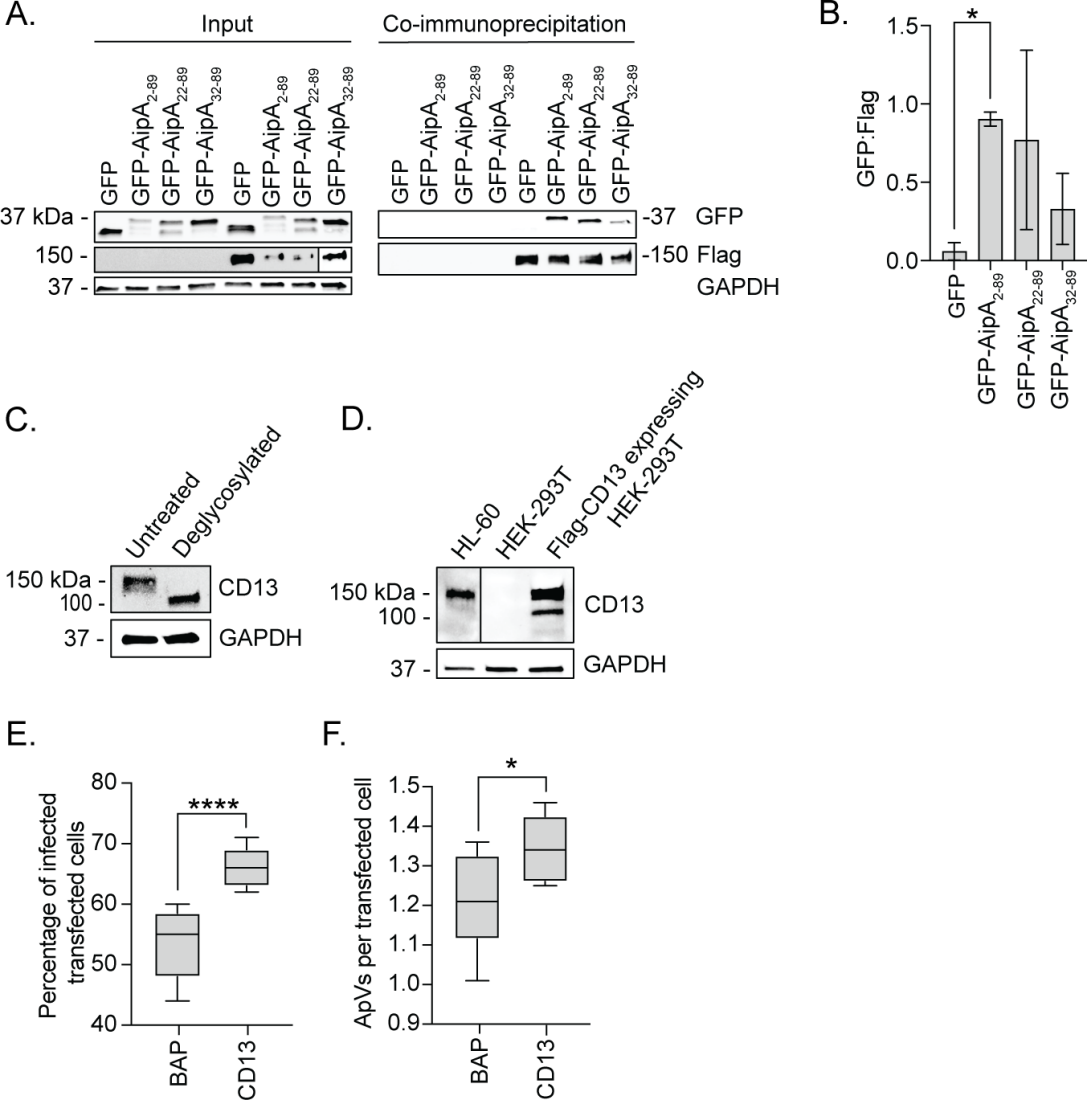
CD13 is an AipA interacting partner that benefits *A. phagocytophilum* infection. (**A**) AipA binds CD13. HeLa cells were transfected to express Flag-CD13 and GFP, GFP-AipA_2–89_, GFP-AipA_22–89_, or GFP-AipA_32–89_ and incubated with anti-Flag M2 affinity gel. Samples were analyzed by western blotting using antibodies specific for GFP, Flag, and glyceraldehyde-3-phosphate dehydrogenase (GAPDH). (**B**) The densitometric signals of co-immunoprecipitated GFP-tagged proteins were normalized to those of Flag. (**C**) Confirmation that CD13 is glycosylated in HL-60 cells. Uninfected HL-60 lysates were subjected to deglycosylation with PNGase F or not and analyzed by western blotting. (**D**) Expression of CD13 in HEK-293T cells. Whole-cell lysates from HL-60, HEK-293T, and Flag-CD13-expressing HEK-293T cells were lysed and analyzed by western blotting to assess the presence of CD13. (**E and F**) Ectopic expression of CD13 increases host cell permissiveness to *A. phagocytophilum* infection. HEK-293T cells expressing Flag-BAP or Flag-CD13 were incubated with *A. phagocytophilum* DC organisms. At 24 h, the cells were fixed, immunolabeled with *A. phagocytophilum* P44 and Flag antibodies, and examined by immunofluorescence microscopy to determine the percentage of infected transfected cells (**E**) and the number of ApVs per cell (**F**) in transfected cells. Results are representative of three independent experiments with similar results. Microscopy data are presented as box-and-whisker plots. The horizontal line denotes the median value (50th percentile). The gray boxes contain the 25th to 75th percentiles of the data set. The whiskers extend from the minimum to maximum values. Values beyond the upper and lower bounds are outliers indicated with black dots. One-way analysis of variance (ANOVA) with Tukey’s post hoc test was used to test for significant differences among groups. Student’s *t* test was used to test for a significant difference between pairs. Statistically significant values are indicated (*, *P* < 0.05; ****, *P* < 0.0001).

The AipA receptor is unknown. Yeast two-hybrid screening using the AipA_1–87_ extracellular domain as bait and a prey human leukocyte cDNA library identified CD13, specifically C-terminal amino acids 851–967, as a possible AipA_1–87_ interacting partner (Table S1 in the supplemental material). Of the candidates, CD13 was the most intriguing because it is expressed on neutrophil surfaces, localizes to caveolae, and is a receptor for internalization by other microbes (19, 21, 27–36, 38–41). To validate if the two proteins are capable of interacting, HeLa cells were transfected to express green fluorescent protein (GFP)-tagged AipA_2–89_, GFP-AipA_22–89_, GFP-AipA_32–89,_ or GFP and Flag-CD13, or GFP proteins alone. Input lysates were subjected to immunoprecipitation to recover Flag-CD13 and interacting proteins followed by western blot analysis. Flag-CD13 co-immunoprecipitated GFP-AipA_2–89_ significantly better than GFP and interacted less efficiently with GFP-AipA_22–89_ and GFP-AipA_32–89_ (Fig. 1A and B). Thus, AipA binds CD13 and minimally utilizes amino acids within 2–21 to do so.

### Ectopic expression of CD13 increases host cell permissiveness to *A. phagocytophilum* infection

Glycosylation of CD13, which is critical for its receptor function, increases its apparent molecular weight from 110 to approximately 160 kDa (20, 42, 43). Peptide-N-glycosidase F (PNGase F) was used to treat lysates of HL-60 cells, a promyelocytic line used for studying *A. phagocytophilum*-host cell interactions (5), to validate that CD13 is glycosylated in these cells. Deglycosylated and untreated control HL-60 cell lysates exhibited anti-CD13 immunoreactive bands of approximately 110 and 160 kDa, respectively (Fig. 1C). To define the relevance of CD13 to *A. phagocytophilum* infectivity, HEK-293T cells transfected to express Flag-tagged CD13 or bacterial alkaline phosphatase (BAP) were incubated with *A. phagocytophilum* organisms followed by immunofluorescence microscopy at 24 h to assess infection and the number of ApVs per cell. HEK-293T cells were selected for this purpose because they do not express CD13 and are also non-phagocytic, amenable to transfection, and susceptible to *A. phagocytophilum* infection (23, 44, 45). Both glycosylated and unglycosylated CD13 were expressed by transfected HEK-293T cells (Fig. 1D). Cell surface presentation of glycosylated Flag-CD13 was confirmed by its susceptibility to trypsin digestion when transfected cells were incubated with the enzyme (Fig. S1). Relative to Flag-BAP, ectopic expression of Flag-CD13 conferred a 24% increase in the percentage of infected HEK-293T cells and an 11% increase in the number of ApVs per cell (Fig. 1E and F). Therefore, surface expression of glycosylated CD13 is linked to increased susceptibility to *A. phagocytophilum* infection.

### The CD13 C-terminus, but not its enzymatic activity, is important for *A. phagocytophilum* infection

**Fig 2:**
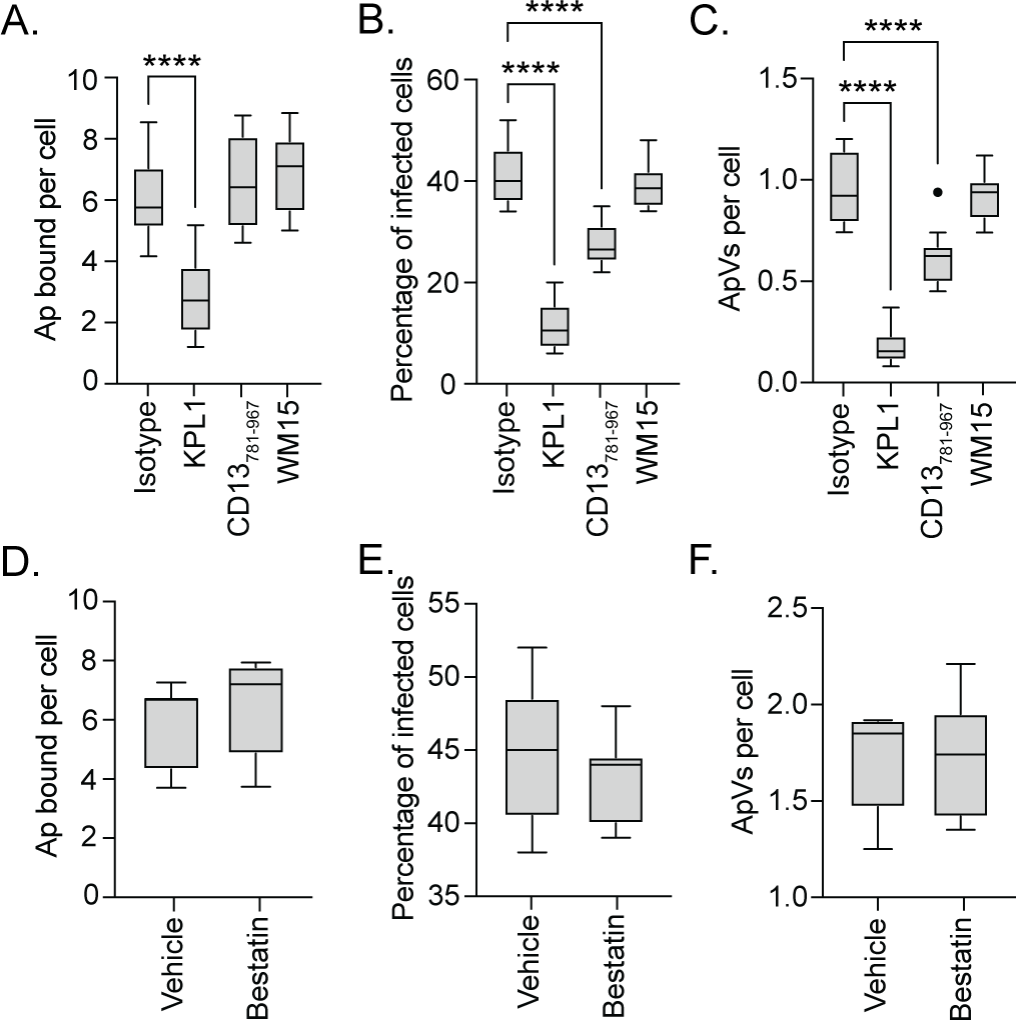
The CD13 C-terminus but not its enzymatic activity is important for *A. phagocytophilum* infection of HL-60 cells. (A–C) HL-60 cells were incubated with antibodies that bind PSGL-1 (KPL1), bind CD13_781–967_, bind the CD13 catalytic domain to inhibit its enzymatic activity (WM15), or isotype control. (D–F) HL-60 cells were treated with 1 mM bestatin or vehicle control (methanol). Treated HL-60 cells were incubated with *A. phagocytophilum* DC organisms. The cells were fixed, immunolabeled with P44 antibody, and analyzed at 1 h to determine the number of *A. phagocytophilum* (Ap) organisms bound per cell (**A and D**). The cells were examined at 24 h to determine the percentage of infected cells (**B and E**) and number of ApVs per cell (**C and F**). Results are indicative of three independent experiments. One-way ANOVA with Tukey’s post hoc test was used to test for significant differences among groups. Student’s *t* test was used to test for a significant difference between pairs. Statistically significant values are indicated (****, *P* < 0.0001).

**Fig 3:**
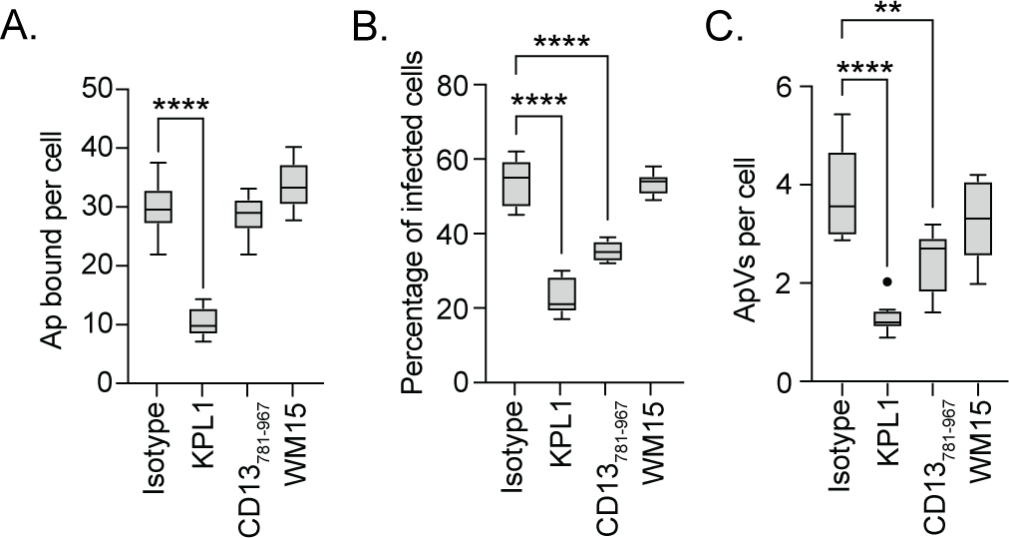
The CD13 C-terminus but not its enzymatic activity is critical for *A. phagocytophilum* infection of human neutrophils. Human neutrophils were treated with the antibodies that bind PSGL-1 (KPL1), bind CD13_781–967_, bind the CD13 catalytic domain to inhibit its enzymatic activity (WM15), or isotype control and subsequently incubated with *A. phagocytophilum* DC organisms. The cells were fixed, immunolabeled with P44 antibody, and analyzed at 1 h to determine the number of *A. phagocytophilum* (Ap) organisms bound per neutrophil (**A**). The cells were examined at 24 h to determine the percentage of infected cells (**B**) and number of ApVs per cell (**C**). Results are indicative of three individual experiments. One-way ANOVA with Tukey’s post hoc test was used to test for a significant difference among groups. Statistically significant values are indicated (**, *P* < 0.01; ****, *P* < 0.0001).

To differentiate whether the *A. phagocytophilum*-CD13 interaction that promotes infection occurs through bacterial engagement of the CD13 C-terminus or if it requires CD13 ectopeptidase activity, promyelocytic HL-60 cells were treated with antibodies against CD13 C-terminal amino acids 781–967 (CD13_781–967_) or the CD13 enzymatic neutralizing antibody, WM15. KPL1, an antibody that blocks *A. phagocytophilum* interaction with the PSGL-1 N-terminus to strongly inhibit bacterial binding and infection (7, 46), served as a positive control. Neither anti-CD13_781–967_ nor WM15 altered *A. phagocytophilum* cellular adherence (Fig. 2A). Relative to their isotype controls, anti-CD13_781–967_ reduced the percentage of infected cells and bacterial load by 33% and 34%, respectively, while WM15 had no effect (Fig. 2B and C). Thus, the interaction between *A. phagocytophilum* and the CD13 C-terminus is important for establishing an optimal infection of HL-60 cells, while CD13 aminopeptidase activity is not. To further confirm that CD13 aminopeptidase activity is not important for *A. phagocytophilum* infection, HL-60 cells were treated with bestatin, an inhibitor of ectopeptidases, or methanol vehicle control and incubated with DC bacteria. The efficacy of bestatin at inhibiting HL-60 cell surface aminopeptidase activity was validated (Fig. S2). Bestatin had no inhibitory effect on *A. phagocytophilum* binding or infection (Fig. 2D through F), confirming that CD13 ectopeptidase activity is dispensable for both. Next, human neutrophils were treated with CD13_781–967_ or WM15 antibodies. While neither inhibited *A. phagocytophilum* cellular adherence, anti-CD13_781–967_ reduced the percentage of infected neutrophils and bacterial load by 35% and 37%, respectively, and WM15 did not (Fig. 3). Therefore, as observed for HL-60 cells, the interaction between *A. phagocytophilum* and the CD13 C-terminus is important for establishing an optimal infection of human neutrophils, while CD13 aminopeptidase activity is not.

### Phosphorylation of Src kinase but not Syk is critical for *A. phagocytophilum* infection

**Fig 4:**
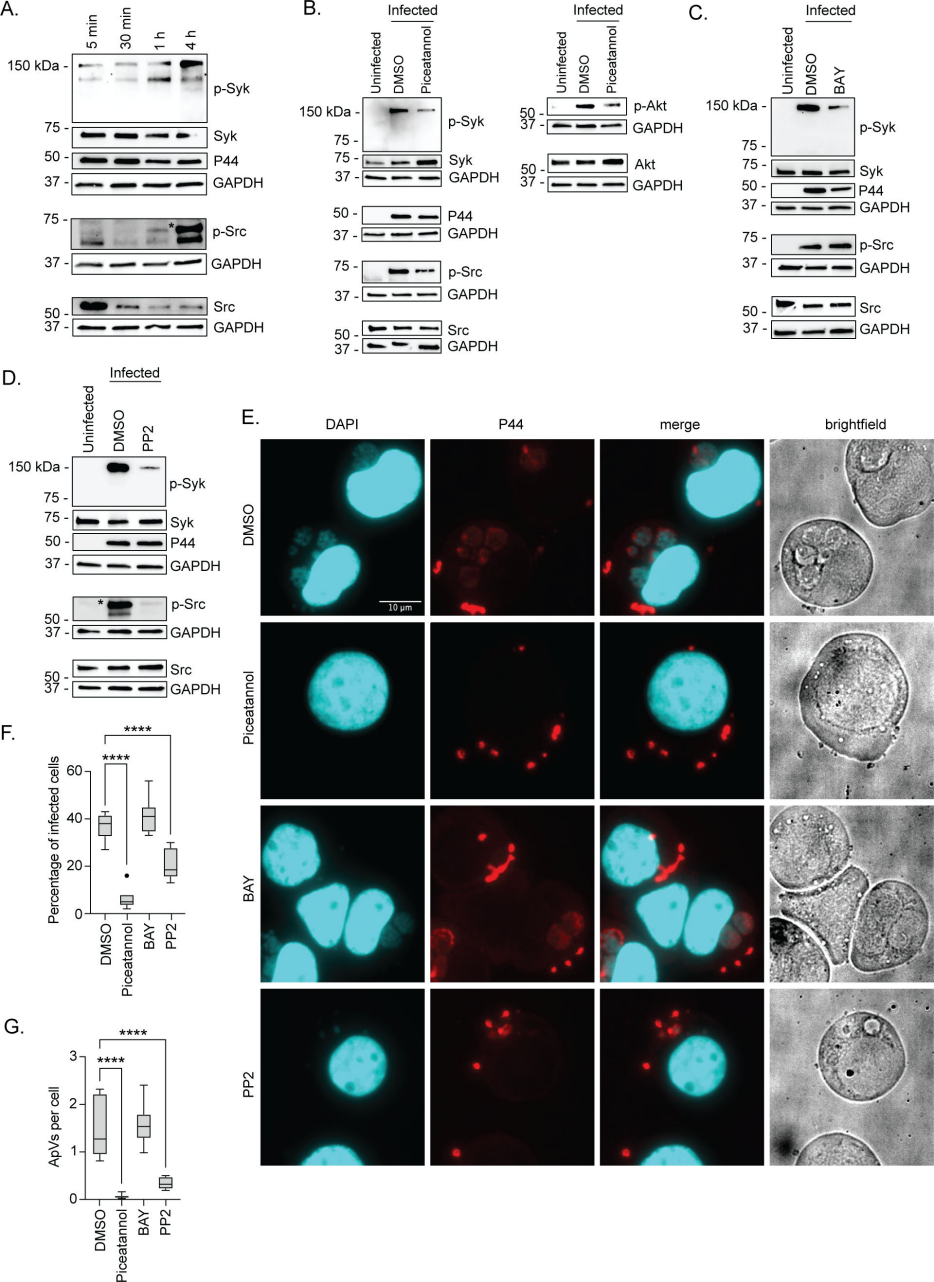
Phosphorylation of Src kinase but not Syk is critical for *A. phagocytophilum* infection. (**A**) HL-60 cells were incubated with *A. phagocytophilum* DC bacteria after which samples were collected at 5 min, 30 min, 1 h, and 4 h. (B–D) HL-60 cells were treated with piceatannol (**B**), BAY (**C**), PP2 (**D**), or vehicle control dimethyl sulfoxide (DMSO) for 1 h and then incubated with DC organisms for 4 h in continued presence of the inhibitor. Samples were subjected to western blotting using antibodies against phospho-Syk (p-Syk; Y525/526) (A–D), Syk (A–D), phospho-Src (p-Src; Y416) (A–D), Src (A–D), phospho-Akt (p-Akt; S473) (**B**), Akt (**B**), *A. phagocytophilum* P44 (A–D), and GAPDH (A–D). Star denotes bands representative of phosphorylated Src. (E–G) HL-60 cells were treated with piceatannol, BAY, PP2, or DMSO and incubated with DC organisms. At 24 h post infection, the cells were fixed and immunolabeled with P44 antibody. Host cell nuclei and bacterial nucleoids were stained with DAPI. The samples were examined by immunofluorescence microscopy. (**E**) Representative merged fluorescent and brightfield micrographs are shown. The percentage of infected cells (**F**) and number of ApVs per cell (**G**) were determined. Results are representative of three independent experiments with similar results. One-way ANOVA with Tukey’s post hoc test was used to test for significant differences among groups. Statistically significant values are indicated (****, *P* < 0.0001).

Since *A. phagocytophilum* exploits CD13 for invasion, we evaluated whether signal transduction elicited when the bacterium engages CD13 promotes infection. Src and Syk are non-receptor tyrosine kinases that become phosphorylated when CD13 is engaged (24, 38). Once activated, they induce signaling events that lead to cytoskeletal rearrangement and endocytosis (47–51). While both kinases have been implicated as being important for *A. phagocytophilum* infection of myeloid cells (15, 52, 53), whether they are indeed activated during bacterial binding and invasion had not been investigated. Accordingly, HL-60 cells were incubated with DC organisms for 4 h, a period required for maximal *A. phagocytophilum* invasion (54–56). Syk and Src phosphorylation during *Anaplasma* uptake were assessed by western blotting at 5 min, 30 min, 1 h, and 4 h. Antibody against the bacterium’s major OMP, P44 (44 kDa protein), was used to confirm infection and assess the overall bacterial burden. Host GAPDH was detected as a loading control. Syk and Src phosphorylation increased over the 4 h duration (Fig. 4A). Multiple bands were observed for phospho-Syk, which is consistent with it being phosphorylated at more than one site (57). Phospho-Src^Tyr416^ antibody detected a band of the expected size as well as an unspecified lower molecular weight receptor tyrosine kinase, the latter of which is an expected result per this antibody’s product sheet and other studies that used it (58, 59). The decrease in band intensity of non-phosphorylated Src and coincident increase in phospho-Src band intensity support the rapid degradation that Src undergoes when phosphorylated (60). Hence, Src and Syk are activated during *A. phagocytophilum* binding and invasion.

Prior studies concluded that Syk was critically important for *A. phagocytophilum* infection based largely on the ability of piceatannol to robustly inhibit infection (15, 16). Although piceatannol is often used as a Syk inhibitor, it has only ~10-fold selectivity for Syk over other kinases, including Src and Akt (61–63). Indeed, when DCs were incubated with piceatannol-treated HL-60 cells, robust inhibition of Syk, Src, and Akt phosphorylation was observed (Fig. 4B). To differentiate between the respective contributions of Syk and Src, the experiment was repeated using Inhibitor IV BAY 61–3606 (BAY) and PP2. BAY is a reversible ATP-competitive inhibitor of Syk that exhibits greater than 600-fold selectivity for it over other kinases (64). PP2 is a reversible Src family kinase-specific inhibitor that prevents phosphorylation of tyrosine 416, an essential step in Src activation (65). BAY inhibited *A. phagocytophilum*-induced phosphorylation of Syk but not Src kinase (Fig. 4C). PP2 nearly abolished Src phosphorylation and robustly inhibited Syk phosphorylation stimulated by the bacterium (Fig. 4D), an observation that agrees with reports that Syk can be phosphorylated directly by Src or indirectly by an immunoreceptor tyrosine-based activation motif-containing intermediate (50, 66).

To evaluate the inhibitors’ effects on *A. phagocytophilum* infection, DC organisms were added to HL-60 cells in the presence of piceatannol, BAY, or PP2. The cells were fixed and permeabilized with methanol followed by immunofluorescence microscopy at 1 h or 24 h to evaluate bacterial adherence and infection, respectively. P44 antibody was used to immunolabel DCs, while 4′,6-diamidino-2-phenylindole (DAPI) was employed to stain host cell nuclei and the nucleoids of bound and intravacuolar bacteria. No treatment impaired *A. phagocytophilum* cellular adherence (Fig. S3). Non-internalized bacteria remained bound at the surfaces of vehicle and inhibitor-treated cells at 24 h (Fig. 4E). Piceatannol reduced the percentage of cells harboring ApVs by 83% and the number of ApVs per cell by 96% (Fig. 4E through G). Strikingly, whereas BAY had no effect, PP2 reduced infection by 44% and ApVs per cell by 77%. Moreover, the ApVs that formed in PP2-treated cells contained single bacteria at 24 h (Fig. 4E), suggesting that Src signaling also benefits *A. phagocytophilum* intravacuolar development post entry. These results clarify that activation of Src kinase but not Syk is essential for *A. phagocytophilum* to optimally infect myeloid cells, and Src phosphorylation both precedes and is critical for the Syk phosphorylation that occurs during *A. phagocytophilum* invasion.

### The AipA-CD13 interaction mediates *A. phagocytophilum* infection by inducing CD13 crosslinking that, in turn, promotes Src kinase and Syk phosphorylation

**Fig 5:**
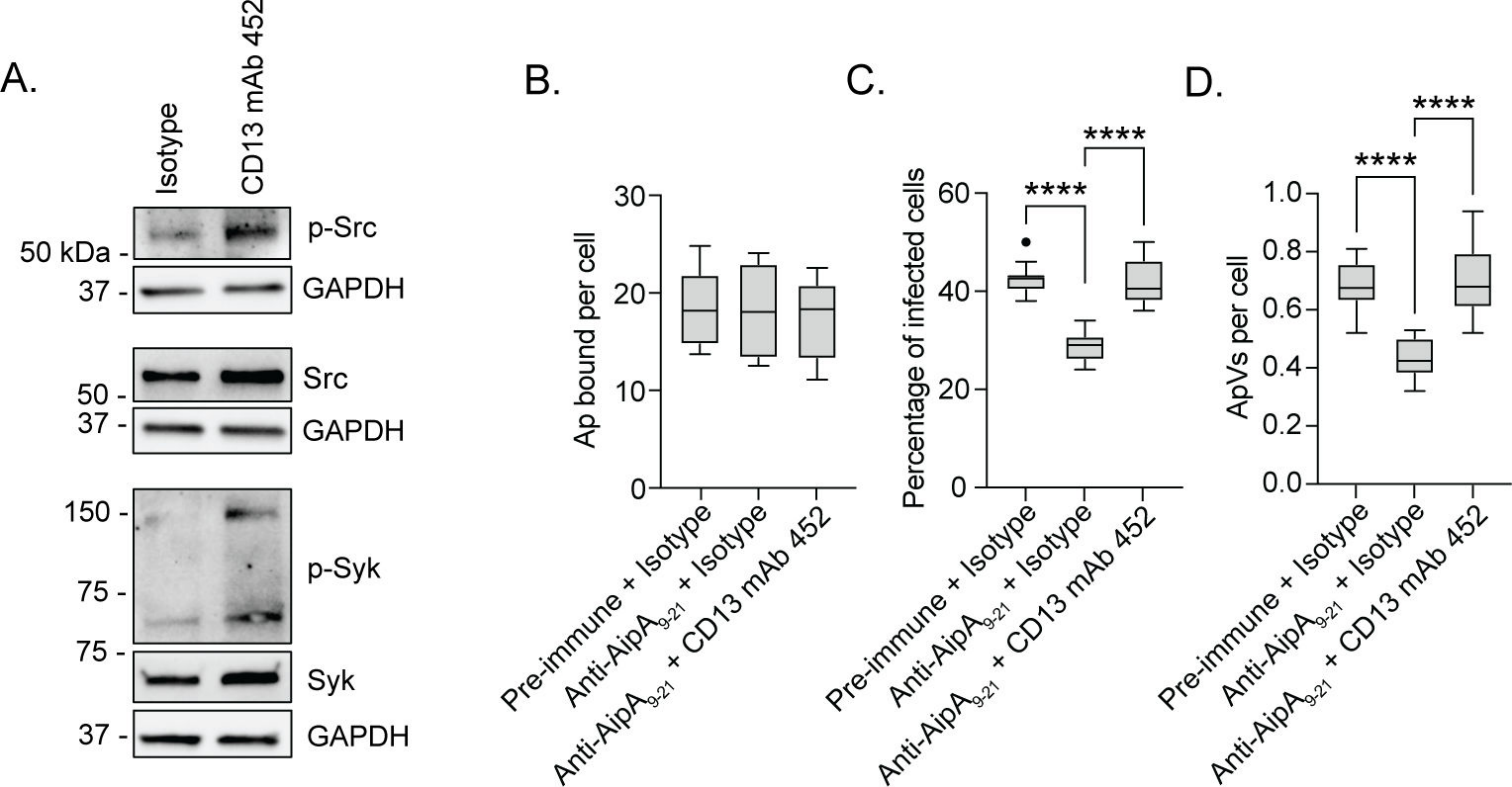
CD13 crosslinking antibody fully restores infectivity of AipA antisera-treated *A. phagocytophilum*. (**A**) Confirmation that CD13 crosslinking mAb 452 increases levels of phospho-Src and phospo-Syk. HL-60 cells were incubated with isotype control or CD13 mAb 452 for 30 min. Samples were analyzed by western blotting using antibodies specific for phospho-Src (Y416), Src, phospho-Syk (Y525/526), Syk, and GAPDH. (B–D) mAb 452 rescues infectivity of AipA_9–21_ antisera-treated *A. phagocytophilum*. DC organisms treated with pre-immune sera or AipA_9–21_ antisera were incubated with HL-60 cells that had been pretreated with isotype control or mAb 452. Cells were analyzed by immunolabeling with *A. phagocytophilum* P44 to determine the number of *A. phagocytophilum* bound per cell at 1 h (**B**), the percentage of infected cells (**C**), and number of ApVs per cell (**D**) at 24 h. Results are representative of four independent experiments. Microscopy data are presented as box-and-whisker plots. One-way ANOVA with Tukey’s post hoc test was used to test for a significant difference among groups. Statistically significant values are indicated (****, *P* < 0.0001).

CD13 engagement activates signal transduction pathways that culminate in endocytosis or phagocytosis (10, 22, 23, 25, 67). To function as a signaling molecule, CD13 must be crosslinked to another CD13 protein by a ligand or anti-CD13 antibody. Indeed, CD13 crosslinking on the plasma membrane by antibodies or viral ligands induces Src and Syk phosphorylation to invoke cytoskeletal changes and promote phagocytic uptake (23, 24, 38). Noncovalent CD13 crosslinking occurs in domain VII that is encompassed by amino acids 581–967 (43) and contains the AipA binding site. To evaluate if the AipA-CD13 interaction promotes *A. phagocytophilum* infection by promoting CD13 crosslinking, HL-60 cells were treated with CD13 crosslinking monoclonal antibody (mAb) 452 or isotype control and incubated with DC organisms that had been pretreated with either AipA_9–21_ antiserum or pre-immune serum. We validated the crosslinking ability of mAb 452 on HL-60 cells by confirming that it stimulated Src and Syk phosphorylation (Fig. 5A). As we previously reported, anti-AipA_9–21_ did not affect bacterial binding but reduced infection by approximately one-third (Fig. 5B through D). In contrast, the competency of AipA_9–21_-treated DC organisms to infect HL-60 cells was fully restored in the presence of mAb 452.

**Fig 6:**
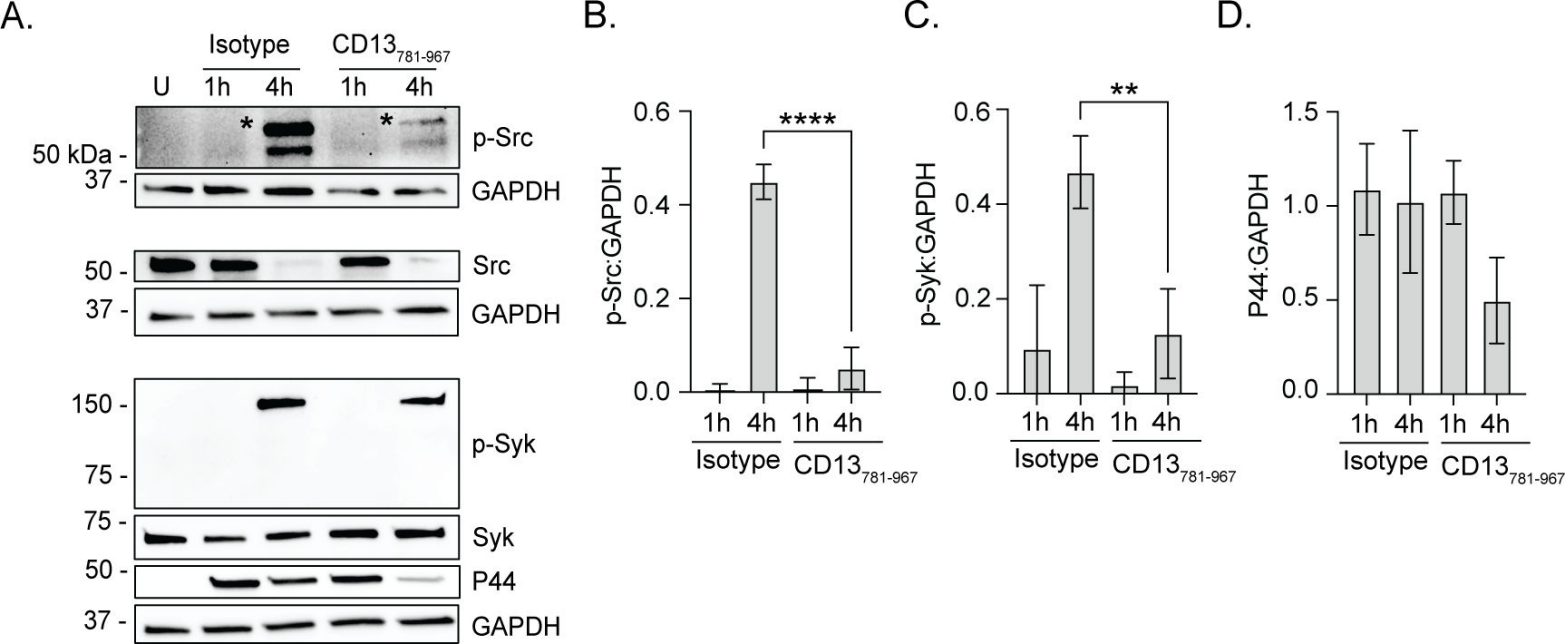
*A. phagocytophilum* engagement of CD13 elicits Src kinase and Syk phosphorylation. HL-60 cells were treated with isotype control or CD13_781–967_ antibodies and then incubated with *A. phagocytophilum* DC organisms. Uninfected HL-60 cells (U) were included as a control. Western blot and densitometric analyses were performed to detect phosphorylated and total levels of Src and Syk at the specified time points. (**A**) Western blots were probed for phospho-Src (Y416), Src, phospho-Syk (Y525/526), Syk, *A. phagocytophilum* P44, and GAPDH. Star indicates bands representative of phosphorylated Src. (B–D) The densitometric signals of phosphorylated Src, Syk, or P44 proteins normalized to that of GAPDH are presented. Data are representative of three independent experiments and presented as the mean ± SD. One-way ANOVA with Tukey’s post hoc test was used to test for significant differences among groups. Statistically significant values relative to isotype-treated cells are indicated (**, *P* < 0.01; ****, *P* < 0.0001).

To directly assess if *A. phagocytophilum* binding to the CD13 C-terminus elicits Src kinase and Syk signaling, HL-60 cells were treated with antibody against CD13_781–967_ or isotype control followed by incubation with DC bacteria. At 4 h, both Src and Syk phosphorylation were significantly reduced when the *A. phagocytoyphilum*-CD13 interaction was blocked (Fig. 6A through C). Additionally, there was an observable but statistically insignificant reduction in P44 immunosignal when the CD13 interaction was blocked, indicating that early-stage infection was partially compromised (Fig. 6A and D). Overall, these data indicate that *A. phagocytophilum* AipA binding to CD13 induces CD13 crosslinking that leads to Src and Syk phosphorylation and is necessary to productively infect myeloid host cells.

### The AipA_9–21_ receptor binding domain is essential for *A. phagocytophilum* to induce Src and Syk phosphorylation

**Fig 7:**
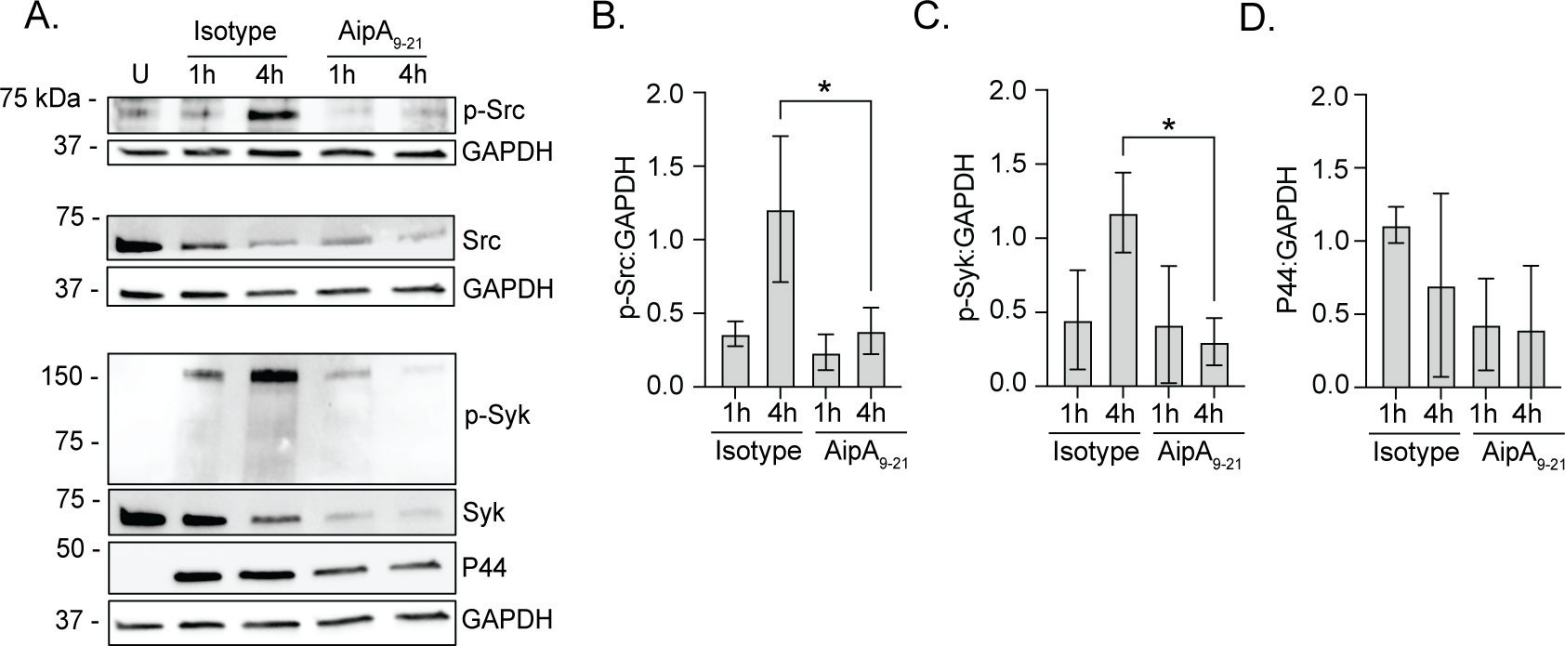
The AipA_9–21_ receptor binding domain is critical for *A. phagocytophilum* to induce Src kinase and Syk phosphorylation. (**A**) Isolated *A. phagocytophilum* DC organisms were treated with AipA_9–21_ antibody or isotype control followed by incubation with HL-60 cells. Uninfected HL-60 cells (U) were included as a control. Western blot and densitometric analyses were performed to detect phosphorylated and total Src and Syk at the specified time points. Western blots were probed for phospho-Src (Y416), Src, phospho-Syk (Y525/526), Syk, *A. phagocytophilum* P44, and GAPDH. (B–D) The densitometric signals of phosphorylated Src, Syk, or P44 proteins were normalized to those of GAPDH. Data are representative of three independent experiments and presented as the mean ± SD. One-way ANOVA with Tukey’s post hoc test was used to test for significant differences among groups. Statistically significant values relative to isotype-treated cells are indicated (*, *P* < 0.05).

To specify if the AipA-CD13 interaction that elicits signal transduction necessary for *A. phagocytophilum* infection requires the AipA_9–21_ receptor binding domain, *A. phagocytophilum* DC organisms were treated with AipA_9–21_ antibody or isotype control and incubated with HL-60 cells. At 4 h, both Src kinase and Syk phosphorylation were nearly abolished, and P44 levels were modestly reduced in anti-AipA_9–21_-treated cells (Fig. 7A through D). Thus, *A. phagocytophilum* utilizes AipA_9–21_ to stimulate Src and Syk signaling during invasion of myeloid host cells.

### CD13 is required for *A. phagocytophilum* to productively infect mice

**Fig 8:**
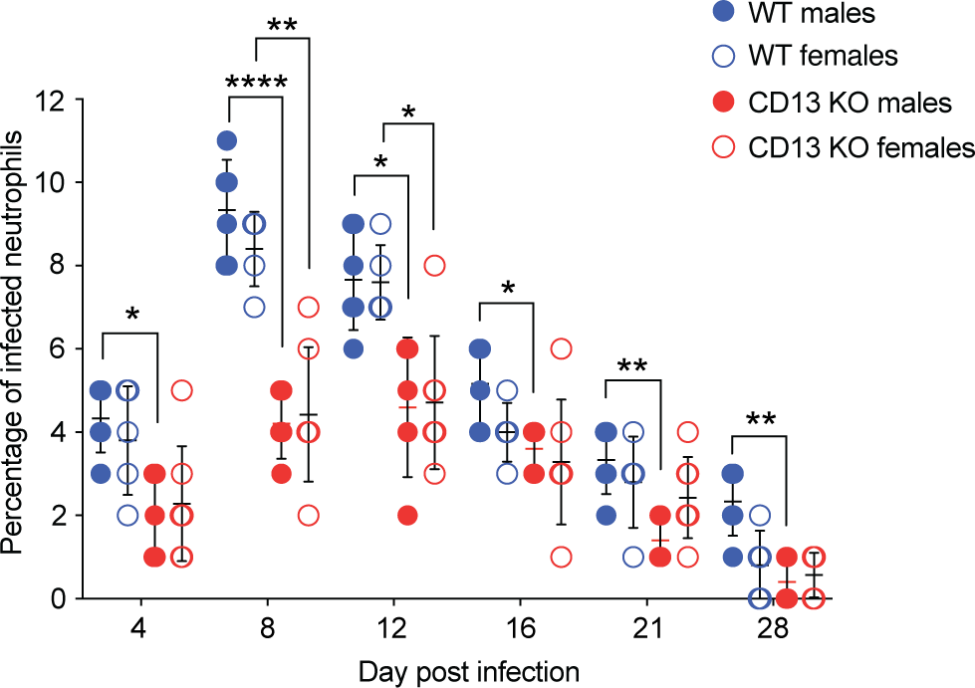
CD13 is required for *A. phagocytophilum* to productively infect mice. CD13 knockout (KO; seven females and five males) or wild-type (WT; five females and six males) mice were intraperitoneally injected with *A. phagocytophilum* DC organisms. Peripheral blood samples drawn on the indicated days were examined by light microscopy for *A. phagocytophilum*-infected neutrophils. Each symbol corresponds to the percentage of infected neutrophils as determined by examining at least 100 neutrophils per mouse. Two-way ANOVA with Tukey’s post hoc test was used to test for significant differences among groups. Data are presented as the mean ± SD. Statistically significant values are indicated (*, *P* < 0.05; **, *P* < 0.01; ****, *P* < 0.0001).

To determine the relevance of CD13 to *A. phagocytophilum* infection *in vivo*, wild-type or CD13 knockout mice were inoculated with DC bacteria. Peripheral blood samples recovered on days 4, 8, 12, 16, 21, and 28 were microscopically examined for neutrophils containing ApVs. Infection in both groups peaked at day 8 and gradually subsided to the lowest detected levels by day 28 (Fig. 8). The percentage of infected neutrophils was lower in CD13 knockout vs wild-type mice at all time points with 2.2- and 1.9-fold differences observed for males and females, respectively, on day 8, the peak of infection. Thus, *A. phagocytophilum* requires CD13 to productively infect neutrophils during mammalian infection.

## DISCUSSION

Our data show that *A. phagocytophilum* AipA binding to CD13 facilitates bacterial uptake and establishes the receptor’s importance to *A. phagocytophilum* infection *in vivo*. AipA_9–21_ binds CD13 within domain VII and does not rely on its ectopeptidase activity for invasion, which is reminiscent of how coronavirus spike glycoproteins and CMV mediate infection using CD13 (27, 35, 36, 68). Another parallel is that *A. phagocytophilum* and human coronavirus 229E (HCoV-229E) enter cells through caveolae (12, 29). Moreover, HCoV-229E internalization at caveolae is CD13 dependent (29). Caveolin-1 knockdown and caveolae disruption via plasmalemmal cholesterol depletion pronouncedly reduce infection by both microorganisms (12, 29). *A. phagocytophilum*, HCoV-229E, and other viruses convergently evolved to exploit CD13 as a portal for entry likely because of the capacity of CD13 to robustly elicit signal transduction and cytoskeletal rearrangement when engaged (19, 22–26). Indeed, when HEK-293T or other non-phagocytic cell lines are transfected for CD13 surface expression, they become phagocytic and significantly more permissive to *A. phagocytophilum* or coronavirus uptake (23, 31). Phagocytosis mediated by CD13 ligation on neutrophils and other cells induces the production of bactericidal reactive oxygen species (23, 38). Consistent with this, *A. phagocytophilum* binding stimulates nicotinamide adenine dinucleotide phosphate (NADPH) oxidase assembly on the plasma membranes of neutrophils and HL-60 cells (55, 56, 69). Yet, it rapidly scavenges exogenously released O_2_^−^ to protect itself while bound and during invasion into an MVB-like vacuole that excludes NADPH oxidase (14, 55, 56, 69). Thus, the bacterium evolved to both exploit CD13 as a receptor for entry and subvert a powerful antimicrobial defense mechanism invoked by CD13 crosslinking.

**Fig 9:**
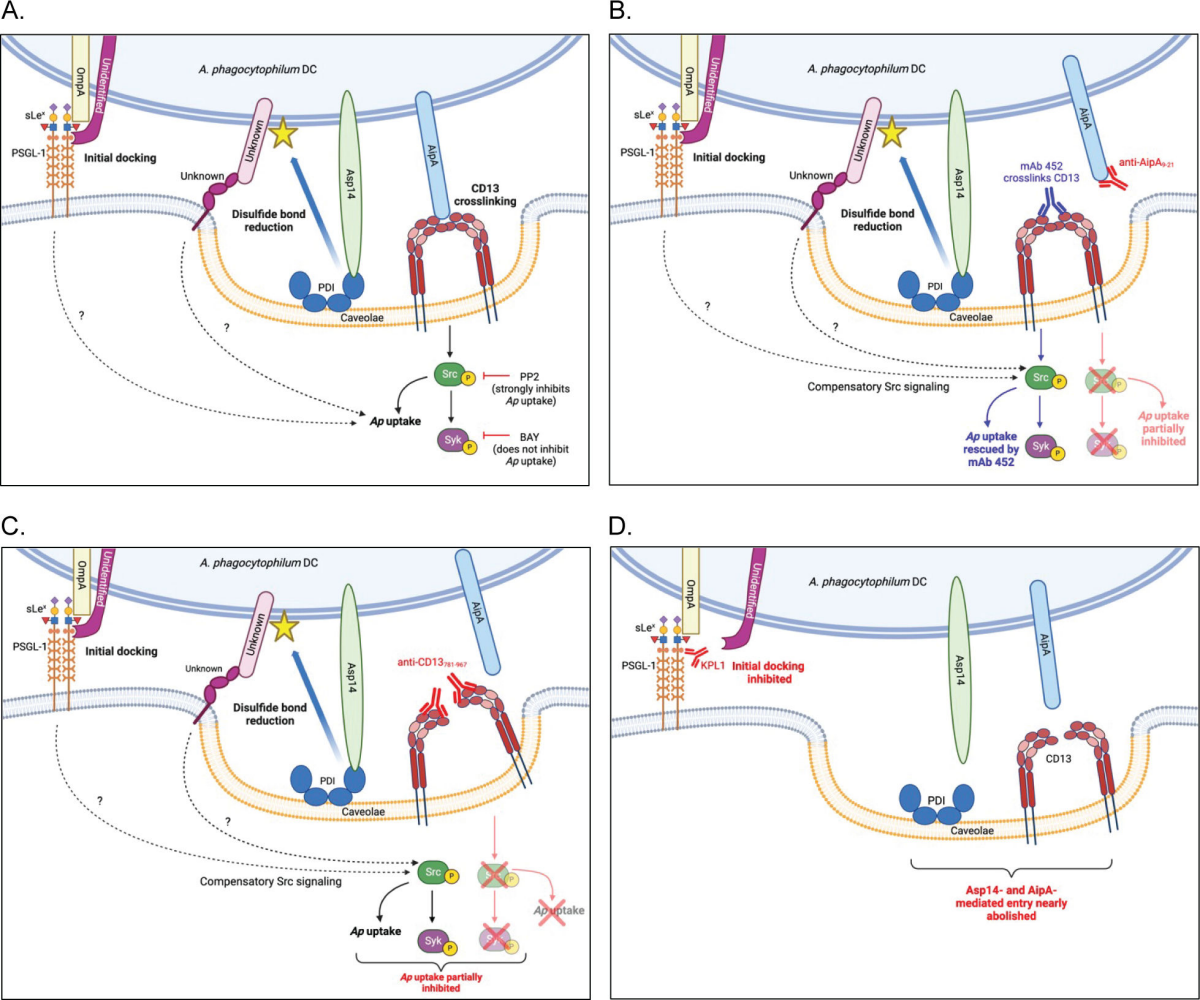
Working model of how the AipA-CD13 interaction contributes to *A. phagocytophilum* infection of host cells. The postulated mechanism by which AipA-CD13 and other *A. phagocytophilum* OMP-host cell receptor interactions mediate infection is based on findings presented herein and in references (4–12). (**A**) *A. phagocytophilum* engages sLe^x^-capped PSGL-1 using OmpA and an unidentified OMP followed by AipA and Asp14 binding to CD13 and PDI, respectively. Together these interactions lead to phosphorylation of Src kinase followed by phospho-Src-dependent phosphorylation of Syk. Signaling through Src but not Syk promotes *A. phagocytophilum* infection, which can be inhibited by PP2 but not BAY. The AipA-CD13-induced Src phosphorylation and downstream events identified in this study are indicated by solid line arrows. Activation of Src via AipA- and CD13-independent interactions inferred from this study is indicated by dashed arrows. The *A. phagocytophilum* OMP that becomes thiol-reduced (star) by PDI and the host cell receptor that it subsequently binds are unknown. (**B**) AipA_9–21_ antibody blocks AipA binding to CD13 to prevent CD13-mediated Src signaling from promoting *A. phagocytophilum* uptake. Because other *A. phagocytophilum*-host cell receptor interactions also induce Src signaling, Src-dependent infection is compromised but not abolished. The addition of mAb 452, which crosslinks CD13 monomers to promote Src signaling *in trans*, restores the ability of anti-AipA_9–21_-treated *A. phagocytophilum* to infect. (**C**) CD13_781–967_ antibody binds the CD13 C-terminus to prevent *A. phagocytophilum* from crosslinking CD13 and thereby inhibit CD13-mediated Src signaling and bacterial uptake. Here again, CD13- and AipA-independent *A. phagocytophilum*-host cell interactions that also activate Src would facilitate infection, albeit at a reduced efficiency. (**D**) KPL1 antibody binds and prevents *A. phagocytophilum* interaction with the PSGL-1 N-terminus. The OmpA-sLe^x^ interaction alone is insufficient to dock the bacterium. KPL1 therefore robustly precludes AipA-CD13, Asp14-PDI, and any other host cell-pathogen interactions from forming to severely impair Src signaling and *A. phagocytophilum* uptake.

*A. phagocytophilum* binding to CD13 via AipA_9–21_ activates Src, which, in turn, is critical for invasion. This argument is supported by the abilities of Src-specific inhibitor PP2, CD13_781–967_ antibody, and Aip_9–21_ antibody to strongly impair *A. phagocytophilum*-induced Src signaling and infection (Fig. 9A through C). It is further supported by the efficacy by which CD13 crosslinking mAb 452 restores *Anaplasma* infectivity in the presence of anti-AipA_9–21_. Interestingly, the ability of mAb 452 to rescue infectivity of anti-AipA_9–21_-coated DC organisms by promoting Src phosphorylation *in trans* indicates that *A. phagocytophilum* does not have to be bound specifically to CD13 for Src signaling to prompt uptake. Perhaps it engages a separate unknown receptor that elicits Src-mediated internalization. Another plausibility is that DCs docked to sLe^x^-capped PSGL-1 can be taken up only after Src becomes phosphorylated (Fig. 9B). In support of this premise, *A. phagocytophilum* DCs bind but cannot infect nonphagocytic Chinese hamster ovary cells transfected to express sLe^x^-capped PSGL-1 (15, 16), suggesting that one or more accessory molecules is needed for entry.

Prior to this study, it had been misperceived by us and others based on findings obtained using piceatannol that *A. phagocytophilum* binding to PSGL-1 activates Syk to facilitate infection (15, 16). Our refined approaches herein elucidated that the AipA-CD13 interaction induces Src phosphorylation that promotes *A. phagocytophilum* uptake and accounts for most of the downstream Syk phosphorylation observed during bacterial entry. Yet, despite its robust activation, Syk is dispensable for *A. phagocytophilum* infection. What, if any, role might Syk play here? Like that shown for B-cell receptor internalization (70), Syk could cluster *A. phagocytophilum* receptors for Src-dependent uptake even though Syk itself is not critical for entry. Few other studies have examined host cell signaling during *A. phagocytophilum* infection of mammalian cells, each of which did so at 1-day post infection or later and hence offer no insight into bacterial entry (17, 71, 72). Nonetheless, the kinases shown to be active during infection or whose inhibition lowered the *Anaplasma* load, PI3K/Akt and Erk1/2 mitogen-activated protein kinase, are downstream from Src in signal transduction pathways (17, 19, 71, 72). Further examination showed that *A. phagocytophilum* internalization is a prerequisite for Erk1/2 activation (17), which fits with Src being activated as part of the pathogen’s internalization process. This collective information supports Src being a lynchpin kinase for orchestrating *A. phagocytophilum* infection of myeloid cells.

Src activated by AipA binding to CD13 could influence the interplay between the *A. phagocytophilum* type 4 secretion system effector, AnkA (73), and the host cell. AnkA is a well-documented nucleomodulin that translocates to the nucleus to epigenetically rewire host cell gene expression into a promicrobial profile (74–79). Upon engaging sLe^x^-capped PSGL-1 on the host cell surface, *A. phagocytophilum* delivers AnkA into the cytosol where it is rapidly tyrosine-phosphorylated by Src and Abeleson (Abl) family tyrosine kinases (15, 52, 73). Src-mediated phosphorylation of AnkA is necessary for it to interact with SHP-1 (Src homology region 2 domain-containing phosphatase 1) (52). Although the outcomes of AnkA phosphorylation beyond SHP-1 binding were not explored further, Abl kinase inhibition using Gleevec by Lin et al. (73) and Src inhibition using PP2 by us herein significantly reduce the bacterial load. PP2’s pronounced negative effect on infection could stem from its ability to inhibit both Src-dependent *A. phagocytophilum* uptake and Src phosphorylation of AnkA. Contrary to our results, Lin et al*.* concluded that PP2 had no effect on *A. phagocytophilum* infection based on their interpretation that the P44 western blot immunosignal strength was not lower for PP2-treated cells compared to vehicle-treated controls, which were their only measure of infection (73). Importantly, we also observed this result but attribute it to *A. phagocytophilum* DC organisms bound at the surfaces of PP2-treated cells as verified by immunofluorescence microscopy. Direct imaging also enabled us to confirm that PP2 reduced the ApV load by 77% and impaired intravacuolar development of the few *A. phagocytophilum* organisms that had been internalized.

When the findings of this and previous investigations are considered, a hierarchy of the *A. phagocytophilum* adhesin/invasin-receptor interactions that facilitate infection of human myeloid cells can be postulated. Anti-AipA_9–21_ has no effect on *A. phagocytophilum* adherence but inhibits Src and Syk phosphorylation to partially reduce infection. Here, the bacterium’s interactions with sLe^x^-capped PSGL-1 and PDI would facilitate binding and entry, respectively (5–8, 10) (Fig. 9B). When the Asp14-cell surface PDI interaction is disrupted by antibodies or PDI is enzymatically inhibited, *A. phagocytophilum* adherence is unaltered, but infection is partially blocked (6, 8, 10). In this scenario, the pathogen would still be able to bind sLe^x^-capped PSGL-1 and induce its Src-mediated uptake via the AipA-CD13 interaction (5, 7, 9–11). Antibody targeting the PSGL-1 N-terminus (Fig. 9D) or sLe^x^ and recombinant OmpA_19–74_ that competitively antagonizes *A. phagocytophilum* access to sLe^x^ strongly inhibits both bacterial binding and infection (5, 7, 9). Thus, *A. phagocytophilum* likely engages sLe^x^-capped PSGL-1 and subsequently forms AipA-CD13 and Asp14-PDI interactions that enable Src-dependent infection (Fig. 9A). This model explains why an antibody cocktail targeting the OmpA, Asp14, and AipA receptor-binding domains most effectively blocks *A. phagocytophilum* infection of HL-60 cells and human neutrophils compared to antibodies targeting any single or any two receptor-binding domains (10). It also supports why immunizing mice against all three binding domains yields anti-AipA and anti-Asp14 responses that protect against *A. phagocytophilum* challenge. The OmpA binding domain turned out to be non-immunogenic. Importantly, mice immunized against only the AipA- or Asp14-binding domain exhibit protection comparable to that observed for mice immunized against both domains (18). Furthermore, AipA_9–21_-immunized (18) and CD13 knockout mice similarly resist *A. phagocytophilum* challenge, underscoring the AipA-CD13 interaction’s key contribution to infection *in vivo*.

Dissecting how pathogenic microbes mediate infection through CD13 has been lauded as an opportunity for developing protective measures against the diseases that they cause (32–35, 68). Likewise, inhibitors of Src, Syk, and other kinases have been cited for their ability to impede *Leishmania amazonensis* uptake (80, 81). Overall, we determined that *A. phagocytophilum* uses AipA, specifically amino acids 9–21, to engage CD13 within residues 781–967 to elicit Src signaling that is vital for it to invade myeloid cells *in vitro* and *in vivo*. Because of the near essentiality of Src activation to *A. phagocytophilum* infection, AipA_9–21_ would be a key immunogen to include in a vaccine against granulocytic anaplasmosis and tickborne fever, while Src could be targeted as host-directed therapeutic against these or other infections that rely on the kinase for infection.

## MATERIALS AND METHODS

### Cell lines and cultivation of *A. phagocytophilum*

HeLa human cervical epithelial cells (CCL-2; ATCC) were cultured in Roswell Park Memorial Institute 1640 medium (Gibco) supplemented with 10% fetal bovine serum (FBS; Gemini Bio-Products). HEK-293T cells (CRL-3216; American Type Culture Collection [ATCC]; Manassas, VA) and uninfected and *A. phagocytophilum* NCH-1 strain-infected RF/6A rhesus monkey choroidal endothelial (CRL-1780; ATCC); and human promyelocytic HL-60 cells (CCL-240; ATCC) were cultured as described previously (14, 44, 82). The *A. phagocytophilum* NCH-1 strain was originally isolated from a patient in Nantucket, MA (83).

### Yeast two-hybrid analysis

ULTImate yeast two-hybrid analysis was conducted by Hybrigenics Services. Mammalian-codon-optimized DNA sequence encoding *A. phagocytophilum* AipA (EPHNCH_1245 in NCH-1 strain genome [https://www.ncbi.nlm.nih.gov/protein/KJV63034.1]; APH_0915 in HZ strain genome [https://www.ncbi.nlm.nih.gov/protein/88598387]) amino acids 1–87 cloned into pB29 as a C-terminal fusion with LexA (N- AipA_1–87_-LexA) or into pB43 as a C-terminal fusion with GAL4 (N-AipA_1–87_-GAL4). Each construct was introduced into yeast as bait and screened by mating with yeast bearing a randomly primed human leukocyte cDNA library (prey). Prey fragments from positively selected clones were PCR amplified, sequenced, and identified using the NCBI GenBank Database (https://www.ncbi.nlm.nih.gov/nucleotide/) and Basic Local Alignment Search Tool (https://blast.ncbi.nlm.nih.gov/Blast.cgi). The predicted biological score was calculated to assess the reliability of each interaction, ranging from the highest (A score) to the lowest (E score) probability of specificity between two proteins (84).

### Mammalian cell transfections and pull-down assays

Mammalian codon-optimized DNA sequences encoding AipA_2–89_, AipA_22–89_, and AipA_32–89_ were synthesized by Genewiz in a pUC57-Kan vector flanked by EcoRI and BamHI restriction sites. Restriction digestion followed by ligation was used to subclone the inserts into pEGFP_C1 vector as previously described (85) to yield pGFP-AipA_2–89_, pGFP-AipA_22–89_, or pGFP-AipA_32–89_. In-frame start and stop codons were introduced using the Takara Bio USA In-Fusion Mutagenesis protocol and pGFP-AipA_2–89_ or pGFP-AipA_22–89_ as template. Primers used to introduce mutations designed using the In-Fusion Cloning Primer Design Tool v1.0 (https://www.takarabio.com/) were Frame shift GFP-AipA_2–89_-F (5′-TCGAATTCCCTGAGCTTCACCATGAGCAAGC-3′), Frame shift GFP-AipA_2–89_-R (5′-GCTCAGGGAATTCGAAGCTTGAGCTCG-3′), Stop codon GFP-AipA_2–89_-F (5′-GCGGATCCTAACTGATCATAATCAGCCATACCA-3′), Stop codon GFP-AipA_2–89_-R (5′-TCAGTTAGGATCCGCCCAGCATTCT-3′), Start codon GFP-AipA_2–89_-F (5′-CCGAATTCATGAGCTTCACCATGAGCAAGC-3′), Start codon GFP-AipA_2–89_-R (5′-AGCTCATGAATTCGGATCCGCCCAGC-3′), Frame shift GFP-AipA_22–89_-F (5′-TCGAATTCCATCGCTTGTAGCATCTTCGACATG-3′), Frame shift GFP-AipA_22–89_-R (5′-AGCGATGGAATTCGAAGCTTGAGCTCGAGATC-3′), and Frame shift GFP-AipA_32–89_-F (5′-GAATTCCCTGGGCGTGAAGAGCACCGCC-3′), and Frame shift GFP-AipA_32–89_-R (5′-TCTTCACGCCCAGGGAATTCGAAGCTTGAGCTCG-3′). HeLa cells grown to 90%–100% confluency in 25 cm^2^ flasks were transfected with 1 µg of pEGFP_C1 or 4 µg of pGFP-AipA_2–89_, pGFP-AipA_22–89_, or pGFP-AipA_32–89_, and/or 4 µg of plasmid DNA encoding Flag-tagged human CD13 (Sino Biological) using Lipofectamine 2000 (Invitrogen) following the manufacturer’s instructions. The cells were incubated at 37°C in a humidified incubator with 5% CO_2_ for 24 h. Spent media was removed, and cells were washed with phosphate-buffered saline (PBS; 1.05 mM KH_2_PO_4_, 155 mM NaCl, 2.96 mM Na_2_HPO_4_, pH 7.4) and collected by scraping. Cells were lysed on ice using immunoprecipitation lysis buffer (50 mM Tris base, 400 nM NaCl, 1 mM EDTA, and 1% Triton X-100) for 45 min. Protein concentrations were determined by Bradford assay. To preclear lysates, protein A/G agarose (Pierce Protein Biology) was washed twice in immunoprecipitation lysis buffer, centrifuged at 8,600 × *g* for 30 s, and added to 400 µg lysates in a final volume of 300 µL. Samples were incubated on a rotator at 4°C for 2–4 h followed by centrifugation at 8,600 × *g* for 30 s. Supernatants were added to anti-Flag M2 affinity gel (Sigma Aldrich) that had been washed twice with immunoprecipitation lysis buffer. Samples were rotated at 4°C overnight followed by centrifugation at 8,600 × *g* for 30 s. Then, 500 µL of immunoprecipitation lysis buffer was added to each sample and rotated for 10 min at room temperature followed by five washes with immunoprecipitation buffer and centrifugation at 8,600 × *g* for 30 s. Washed beads were resuspended in Laemmli buffer (Bio-Rad) with 500 mM imidazole (Thermo Fisher Scientific) and incubated at 100°C for 10 min to elute-bound proteins. Input lysates (30 µg) and eluates were resolved by SDS-PAGE and screened by western blotting.

### Infection assays

*A. phagocytophilum*-infected HL-60 cells were centrifuged at 5,200 × *g* for 15 min. The cell pellet was resuspended in 6 mL of PBS followed by sonication using a Misonix-4000 sonicator (amplitude 30, four 8 s pulses interrupted by 8 s intervals) to leave only DC bacteria intact. The sonicate was subjected to differential centrifugation to remove host cell debris by a single centrifugation at 750 × *g* for 5 min followed by centrifugation twice at 1,000 × *g* for 5 min. DC organisms were harvested by a final 10 min spin of 5,200 × *g* and resuspended in Iscove’s modified Dulbecco’s medium (IMDM; Gibco) supplemented with 10% (vol/vol) FBS (Gemini Bio-Products) (IMDM-10). Uninfected HL-60 cells were incubated with DC organisms for 1 h in a humidified incubator at 37°C with 5% CO_2_ and inverted every 10 min. Cells were washed 2–3 times with PBS to remove residual unbound bacteria then plated in IMDM-10 in a humidified incubator at 37°C with 5% CO_2_. For immunofluorescence assays, 0.25 × 10^6^ HL-60 cells or neutrophils were used per condition, while for western blotting, 1.5 × 10^6^ HL-60 cells were used per condition. In each case, HL-60 cells were incubated with DC bacteria recovered from two to four times as many infected HL-60 cells. For the AipA_9–21_ antibody blocking assays to evaluate cellular signaling outlined below, the DC isolation protocol was modified as follows. *A. phagocytophilum* infected HL-60 cells were centrifuged at 500 × *g* for 5 min to pellet host cells, leaving infectious DC organisms in the supernatant. The supernatant was transferred to a new conical and sonicated for 8 s at amplitude 30 to break up residual host cell debris and then centrifuged at 5,200 × *g* for 10 min to pellet DC organisms, which were resuspended in IMDM-10 and incubated with naïve HL-60 cells. To evaluate the relevance of CD13 to *A. phagocytophilum* infection, HEK-293T cells seeded on coverslips in 24-well plates were transfected with 0.4 µg of pFlag-BAP (Sigma-Aldrich) or plasmid encoding Flag-tagged human CD13 (Sino Biological). At 18 h, the cells were incubated with *A. phagocytophilum* DC bacteria that had been naturally released from RF/6A cells into the culture media as previously described (45). Surface expression of Flag-CD13 on HEK-293T cells was verified by incubation of transfected cells with 0.05% trypsin (Gibco) or PBS for 10 min at 37°C. Samples were analyzed by western blotting.

### Antibody inhibition of *A. phagocytophilum* infection

To determine the relevance of CD13 to *A. phagocytophilum* binding and infection of host cells, inhibition assays utilizing CD13 antibodies were performed. HL-60 cells were treated with 10 µg/mL of mouse IgG1 isotype control (Invitrogen [02–6100]), purified mouse anti-human CD162 (PSGL-1; Clone KPL-1; BD Biosciences [556053]), CD13 mAb 1C7D7 (CD13_781–967_; Invitrogen [MA1-181]), or CD13 antibody WM15 (Bio-Rad [MCA1270]) for 1 h during which the tubes were inverted every 10 min. The cells were then incubated with *A. phagocytophilum* DC organisms isolated from twice as many infected HL-60 cells. To evaluate the relevance of CD13 crosslinking to AipA-mediated *A. phagocytophilum* infection, isolated DC bacteria from four times as many infected HL-60 cells were incubated with 100 µg/mL of heat-inactivated rabbit AipA_9–21_ antiserum (10) or rabbit pre-immune serum for 1 h prior to incubation with HL-60 cells in the presence of 10 µg/mL mouse IgG1 isotype control (Invitrogen [02-6100]) or anti-CD13 clone 452 mAb (Millipore Sigma [Q3121710]) for 1 h. To evaluate cellular signaling while inhibiting the AipA-CD13 interaction, HL-60 cells were incubated with isolated *A. phagocytophilum* DC organisms from twice as many infected HL-60 cells. HL-60 cells were treated with mouse IgG1 or CD13 mAb 1C7D7. Alternatively, DC organisms were treated with rabbit IgG (Invitrogen [02-6102]) or rabbit anti-AipA_9–21_ for 1 h at 37°C (10). Samples were collected at 1 and 4 h post infection for western blot analysis. To prevent cross-reactivity of the secondary antibody with the anti-AipA_9–21_ heavy chain during western blot analyses, samples were incubated in 0.05% trypsin (Gibco) for 10 min at 37°C to remove blocking antibody bound to *A. phagocytophilum* adhered to the HL-60 cell surfaces.

### Neutrophil infections

Human neutrophils were isolated as described previously (86). The viability and purity of neutrophils were verified by trypan blue exclusion and Diff-Quick staining of cytocentrifuged slides. Neutrophils were incubated with isolated *A. phagocytophilum* DC organisms from four times as many infected HL-60 cells.

### Indirect immunofluorescence microscopy

*A. phagocytophilum*-infected HL-60 cells (2 × 10^4^) were cytocentrifuged onto glass slides at 1,000 RCF for 3 min in a Shandon Cytospin 4 centrifuge (Thermo Electron) followed by fixation and permeabilization in Hema3 fixative (Thermo Fisher Scientific) for 5–10 min. *A. phagocytophilum* P44 was detected by sequentially staining slides for 1 h each with P44 antiserum (14) (1:500) and Alexa Fluorochrome 594-conjugated chicken anti-rabbit IgG (1:1,000; Invitrogen [A21442]). *A. phagocytophilum*-infected HEK-293T cells on coverslips were fixed in 4% paraformaldehyde and permeabilized with 0.5% Triton X-100 at 24 h post infection. P44 and Flag-CD13 were detected by sequentially staining with P44 antiserum and monoclonal anti-Flag M2 antibody (1:1,000; Sigma-Aldrich [F1804]) followed by Alexa Fluorochrome 594-conjugated chicken anti-rabbit IgG (1:1,000; Invitrogen [A21442]) and Alexa Fluorochrome 488-conjugated goat anti-mouse IgG (1:1,000; Invitrogen [A11001]), respectively. Coverslips were mounted with ProLong Gold Antifade mounting media plus DAPI (Invitrogen). To determine the number of *A. phagocytophilum* bound per cell, 50 cells per slide were evaluated at 1 h post infection using an Olympus BX51 spinning disk confocal microscope (Olympus, Shinjuku City, Tokyo, Japan). To determine the percentage of infected cells at 24 h post infection, 100 cells were evaluated for ApVs, and the number of ApVs per cell was determined by dividing the total number of ApVs by 100. Micrographs were acquired via epifluorescence and brightfield microscopy using a Leica DMi8 inverted microscope affixed with the following Leica package: Leica EL6000 lamp at 460 nm and 630 nm and band-pass filters at 420/30 nm and 570/20 nm. Image acquisition was performed with an Andor iXon Ultra 888 EMCCD camera (Oxford Instruments) and a 63× water-immersion objective with 1.2 numeric aperture. Image processing was performed using the Leica LAS X software and ImageJ Fiji (87).

### Western blotting

Proteins were extracted from uninfected and infected HL-60 cells and HEK-293T cells at the indicated time points using radioimmunoprecipitation assay buffer (50 mM Tris-HCl [pH 7.4], 150 mM NaCl, 1% NP-40, 1% sodium deoxycholate, and 1 mM EDTA [pH 8]) supplemented with Halt Protease and Phosphatase Inhibitor Cocktail (Thermo Fisher Scientific; 1:100). In some cases, HL-60 cell lysates were subjected to deglycosylation treatment using PNGase F (New England Biolabs; Ipswich, MA) following the manufacturer’s instructions prior to western blot analysis as a means for confirming CD13 glycosylation. To verify that CD13 mAb 452 elicits Src kinase and Syk signaling, 1 × 10^6^ HL-60 cells were incubated with 1 µg/mL of CD13 clone 452 mAb or mouse IgG1 for 30 min at 37°C, after which samples were collected and analyzed by western blotting. Protein concentrations were determined by Bradford protein assay. Lysates were loaded into 4%–15% reducing SDS-PAGE gels (Bio-Rad) and transferred onto nitrocellulose membranes for western blotting. Blocking, antibody incubation periods, and washing conditions between primary and secondary antibody incubations were as described previously (88). Primary antibody conditions followed the manufacturer’s recommendations and targeted Syk (1:1,000; Cell Signaling Technology [2712]), phospho-Syk (Tyr525/526; C87C1; 1:1,000; Cell Signaling Technology [2710]), Src (36D10; 1:1,000; Cell Signaling Technology [2109]), phospho-Src family (Tyr416; 1:1,000; Cell Signaling Technology [2101]), Akt (pan; C67E7; 1:1,000; Cell Signaling Technology [4691]), phospho-Akt (Ser473; D9E) XP (1:1,000; Cell Signaling Technology [4060]), *A. phagocytophilum* P44 (14) (1:5,000), CD13 (1:500; Abcam [ab154116]), GFP (1:1,000; Thermo Fisher Scientific [A11122]), monoclonal anti-Flag M2 antibody (1:1,000; Sigma-Aldrich [F1804]), or GAPDH (G-9; 1:750; Santa Cruz [sc-365062]). Secondary antibodies were horseradish peroxidase-linked anti-mouse IgG (1:10,000; Cell Signaling Technology [7076S]) or anti-rabbit IgG (1:10,000; Cell Signaling Technology [7074S]). Blots were incubated with chemiluminescent substrate and imaged as described (88). The densitometric signals of GFP, phosphorylated Src kinase, phosphorylated Syk, and P44 obtained using Image Lab 6.1 (BioRad) were normalized to that of either Flag or GAPDH from the same sample.

### Kinase inhibitor studies

Stocks of piceatannol (Sigma-Aldrich), Syk Inhibitor IV BAY 61-3606 (BAY; Millipore Sigma), and PP2 (Sigma-Aldrich) were made in DMSO (Thermo Fisher Scientific) at 10 mg mL^−1^. Bestatin (Sigma-Aldrich) stock was made in methanol (Thermo Fisher Scientific) at 5 mg mL^−1^. Final concentrations of 15 µM piceatannol, 1 µM BAY, 30 µM PP2, and 1 mM bestatin were used in experiments. Vehicle controls were equivalent to volumes of DMSO or methanol alone. HL-60 cells were treated with inhibitors for 1 h followed by incubation with *A. phagocytophilum* DC organisms. Except for bestatin, infection proceeded in the presence of inhibitors. Bestatin enzymatic inhibition of CD13 activity was verified by incubating isolated HL-60 cells with 1 mM bestatin or methanol for 1 h. Both treatment groups received 2 mM L-alanine-4-nitroanilide hydrochloride (Sigma-Aldrich) in a 0.2 M sodium phosphate buffer, pH 7, and placed in a water bath at 40°C for 10 min. Absorbance was read at 340 nm (89).

### Mouse studies

All animal research was performed under the approval of the Institutional Animal Care and Use Committee at Virginia Commonwealth University (Protocol Number AM10220). CD13^−/−^ mice, which were generated on a C57BL/6 background, have been described previously (90). C57BL/6 mice were purchased from Jackson Laboratories (Bar Harbor, ME). Mice were 7 to 9 weeks old when infection experiments were initiated. Total of six wild-type males, five wild-type females, five CD13^−/−^ males, and seven CD13^−/−^ females were intraperitoneally inoculated with 1 × 10^8^ *A. phagocytophilum* DC organisms as described (91–93). Blood was collected from the tail vein on days 0, 4, 8, 12, 16, 21, and 28. Heparin (Sigma-Aldrich) at 100 U mL^−1^ was added to blood samples. Quantification of the peripheral blood *A. phagocytophilum* burden was determined as described (91).

### Statistical analysis

Statistical analyses were performed using the Prism 7.0 software package (GraphPad; San Diego, CA). One-way analysis of variance (ANOVA) and two-way ANOVA with Tukey’s multiple comparisons test were used to test for significant differences among the groups. An unpaired student’s *t*-test was used to test for statistical significance between paired data. Statistical significance was set at *P* values of <0.05.

## SUPPLEMENTAL MATERIAL

Supplemental material mbio.01561-24-s0001.pdf Fig. S1 to S3; Table S1.

ASM does not own the copyrights to Supplemental Material that may be linked to, or accessed through, an article. The authors have granted ASM a non-exclusive, world-wide license to publish the Supplemental Material files. Please contact the corresponding author directly for reuse.
