# Supplementary material for: Anaplasma phagocytophilum invasin AipA interacts with CD13 to elicit Src kinase signaling that promotes infection
Source: mBio. 2024 Sep 26;15(10):e01561-24. doi: 10.1128/mbio.01561-24 (PMC11481542; doi:10.1128/mbio.01561-24)
Supplement: Supplemental material — Fig. S1 to S3; Table S1. [file mbio.01561-24-s0001.pdf]

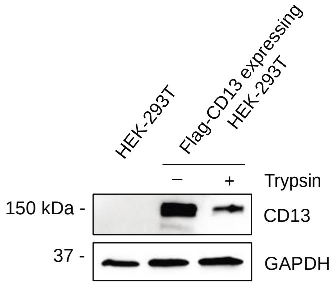

Fig S1. CD13 ectopically expressed by HEK-293T cells is presented on the cell surface. HEK-293T cells transfected to express Flag-CD13 were incubated in the presence or absence of trypsin. Samples were analyzed by Western blotting, probing for CD13 and GAPDH. Lysate of non-transfected HEK-293T cells was included as a control demonstrating the absence of CD13. The data shown is representative of three independent experiments.

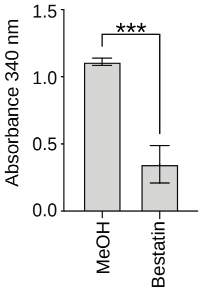

Fig S2. Bestatin inhibits CD13 aminopeptidase activity. HL-60 cells pretreated with bestatin or methanol vehicle control (MeOH) were incubated with a known CD13 substrate, L-alanine 4-nitroanilide-HCl. Absorbance was read at 340 nm. Data are representative of three independent experiments and presented as the mean ± SD. Student's *t* test was used to test for a significant difference between pairs. Statistically significant values are indicated (***, $P < 0.001$).

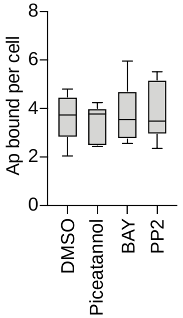

Fig S3. Piceatannol, PP2, and BAY do not affect *A. phagocytophilum* binding to host cells. HL-60 cells were treated with piceatannol, BAY, PP2, or DMSO vehicle control for 1 h and then incubated with *A. phagocytophilum* DC organisms. At 1 h, the cells were immunolabeled with *A. phagocytophilum* P44 antibody and analyzed by immunofluorescence microscopy to determine the number of *A. phagocytophilum* organisms bound per cell. Data are representative of three independent experiments. One-way ANOVA with Tukey's post hoc test was used to test for a significant difference among groups.

Table 1. AipA candidate interacting partners identified via yeast two-hybrid analysis

Candidate	Name and function	Known subcellular localization[a]	PBS[b]
ACTG1	Actin gamma 1; cell motility and cytoskeleton maintenance	C	D
ANPEP	Alanyl aminopeptidase (CD13); release of N-terminal amino acids	PM	B
ASCC2	Activating signal co-integrator 1 complex subunit 2; DNA damage repair	N	D
ASNA1	Guided entry of tail-anchored proteins factor 3; ATPAse	C, N, ER	D
BECN1	Beclin 1; autophagy	C, N, ER, G, M, E	D
CD44	CD44 antigen; cell-cell interactions, cell adhesion and migration	PM	D
COPS5	COP9 signalosome subunit 5; involved in various cellular/developmental processes	C, N	F
COQ5	Coenzyme Q5; Methyltransferase	M	D
GATAD2B	GATA zinc finger domain containing 2B; transcriptional repressor	N	D
HIVEP1	Zinc finger protein 40; transcriptional activator of viral genes	PM, N	D
IL-32β	Interleukin 32; innate and adaptive immune responses	Secreted	D
NEDD9	Neural precursor cell expressed developmentally down-regulated 9; mediates protein-protein interactions for signal transduction pathways	C, N, G	F
PLIN2	Perilipin 2; involved in development and maintenance of adipose tissue	C	B
PSRC2	Zinc finger C3H1-type containing; directs polyadenylated poly(A) RNAs for exosomal degradation	N	C
PTPRC_var1	Protein tyrosine phosphatase receptor type C; cell growth, differentiation, mitosis, and oncogenic transformation	C	D
RABAC1	Rab acceptor 1; vesicle formation from the Golgi complex	PM, C, G	D
RANBP9	RAN binding protein 9; GTP binding protein	N, PM	F
RNF123	Ring finger protein 123; protein-protein and protein-DNA interactions	C	F
RXRA	Retinoid X receptor alpha; transcription factor	N	D
SLC12A6	Solute carrier family 12 member 6; electroneutral potassium-chloride cotransport	M	D
SNAPIN	SNAP associated protein; intracellular vesicle trafficking and synaptic vesicle recycling	L, PM, G	F
ST13	ST13 Hsp70 interacting protein; may contribute to the interaction of HSC70 with various target proteins	C	F
TBP	TATA-box binding protein; functions at the core of the DNA-binding multiprotein factor TFIID	N	D
TRPC4AP	Transient receptor potential cation channel subfamily C member 4 associated protein; adapter of a DCX E3	PM	D

	ubiquitin-protein ligase complex required for cell cycle control		
TTC1	Tetratricopeptide repeat domain 1; protein-protein interactions	C	D
USP34	Ubiquitin specific peptidase 34; regulator of Wnt signaling pathway	C, N	B
XRCC6	X-ray repair cross complementing 6; single-stranded DNA-dependent ATP-dependent helicase	N	E
YIPF5	Yip1 domain family member 5; transport between endoplasmic reticulum and Golgi	G, ER	B

[a]Abbreviated cellular locations: G, Golgi; E, endosome; M, mitochondria; PM, plasma membrane; N, nucleus; ER, endoplasmic reticulum; C, cytosol; L, lysosome. [b]The predicted biological score (PBS) was calculated for each candidate protein to assess the reliability of each predicted interaction, scores range from the highest probability of specificity (score of A) to the lowest probability of specificity (score of F) between two proteins.
